# Synergistic effect of polydopamine–polyethylenimine copolymer coating on graphene oxide for EVA nanocomposites and high-performance triboelectric nanogenerators†

**DOI:** 10.1039/c9na00142e

**Published:** 2019-05-21

**Authors:** Sunanda Roy, Le Van Hai, Jaehwan Kim

**Affiliations:** Mechanical Engineering, Inha University, South Korea 100, Inha-Ro, Nam-Ku Incheon 22212 South Korea corporate00@gmail.com jaehwan@inha.ac.kr

## Abstract

While the demand for lightweight high-strength nanocomposites is immense, their progress has been severely limited due to inferior filler dispersion and filler–matrix interface adhesion. This article reports a novel modification of graphene oxide (GO) encapsulated by the copolymer of polydopamine (PD) and polyethylenimine (PEI) *via* a Michael addition reaction, aiming to create robust ethylene vinyl acetate copolymer (EVA) nanocomposites even at very low amounts of filler loading by overcoming the above hindrances. It has been found that the addition of only 1.2 wt% modified GO (*i.e.*, PD–PEI–rGO) increased the tensile strength, Young's modulus and storage modulus of EVA composites by 80%, 50% and 24%, respectively. These increments surpass many recent claims on relevant composites. Excellent molecular level dispersion was also observed from the fracture surface SEM images. Being amine-rich with high electron-donating capability and mechanically robust, the nanocomposite served as an outstanding tribopositive material, thereby generating 7.49 V and 4.06 μA output voltage and current, respectively, when employed in a triboelectric nanogenerator (TENG). The high electrical outputs led the device to light up 43 blue LEDs instantaneously upon hand pressing, demonstrating that the nanocomposite is indeed a promising candidate for harvesting green energy. Moreover, the nanogenerator displayed outstanding cyclic performance stability (even after 8000 cycles) and environmental durability.

## Abstract 2

The fabrication of flexible high-performance EVA–graphene oxide nanocomposite triboelectric nanogenerators and the lighting of 43 blue LEDs.

## Graphical abstract

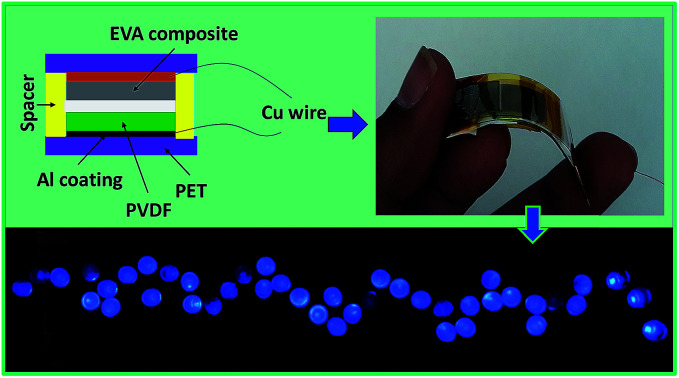

## Introduction

Among a variety of polymer nanocomposites, polymer–carbon nanomaterial composites, especially carbon nanotubes (CNTs) and graphene-based ones, are of the highest priority of current research interests as they deliver high electrical, thermal, mechanical and barrier properties to the composites at significantly low amounts.^1–4^ While graphene is an atomically thick, 2-D sheet material composed of sp^2^ carbon atoms arranged in a honeycomb structure, CNTs are zero-dimensional cylindrically rolled sheets of graphene.^5,6^ Since 2004, the successful production and isolation of graphene^7^ have taken the nanotechnology sector by storm, with exponential growth in its applications. Graphene is regarded as the “magic bullet” for the composite world, owing to its remarkably high mechanical, electrical, thermal conductivity and gas and chemical barrier properties. Despite its huge merits and the large amount of published literature on graphene nanocomposites, scientists are still exploring the hidden potentials of this material in order to expand the scope of its application, because the improved properties achieved by graphene are still far lower than the theoretical predictions. Critical challenges that severely limit the continuous advancement of graphene for structural applications is its poor compatibility, non-uniform dispersion and weak interface adhesion with the polymer matrices due to the lack of adequate surface functionalities.^1,8–10^ Moreover, the strong interlayer van der Waals forces cause the graphene nanosheets to form irreversible agglomerates (or restack), making it extremely difficult to handle. Thus, the prevention of restacking should, therefore, be attained prior to incorporation in any system. Moreover, the level of enhancements has been found to depend significantly on the degree of dispersion, alignment and filler–matrix interface adhesion. Hence, to overcome the above drawbacks and enhance the chemical affinity towards polymer matrices and to successfully harness maximum reinforcement, the surface modification of graphene has become imperative.

**Fig. 1:**
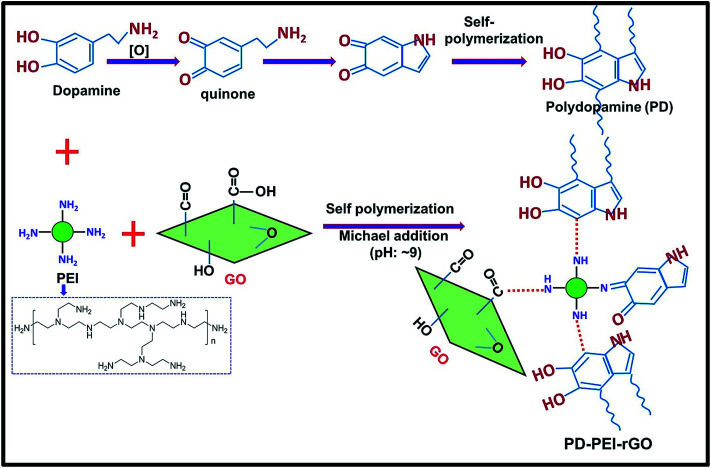
Schematic illustrations of the preparation of PD–PEI–rGO.

Therefore, this article aims herein to design a novel surface modification process for graphene oxide (GO) in order to develop mechanically stronger polymer nanocomposites even at significantly low amounts of loading. GO was selected, as it is the most widely preferred derivative of graphene material, containing various oxygenated functional groups (*e.g.*, hydroxyl, epoxide, carbonyl and carboxylic acid groups)^1,8–10^ that can be used as an anchorage for further surface treatments depending on the needs. Here, ethylene vinyl acetate copolymer (EVA) was chosen as the matrix, since it has widespread applications in numerous areas including paints, adhesives, coatings, textiles, cables, automotive plastic fuel tanks, *etc.*^11–14^ The GO was modified with polyethylenimine (PEI) through the assistance of polydopamine (PD) coating *via* the Michael addition reaction, as demonstrated in Fig. 1. The surface chemistry and structural properties of the modified GO (PD–PEI–rGO) were studied comprehensively. The performance of the composites was evaluated in terms of tensile strength and dynamic mechanical properties. Super reinforcement was achieved by the addition of only 1.2 wt% PD–PEI–rGO, and the values were found to be much higher than most previously reported EVA nanocomposites.

Besides the structural importance, we explored a new potential application of this composite material as a sustainable energy harvesting device, called a triboelectric nanogenerator (TENG). Recently, energy harvesting and conversion devices using lightweight materials have become an emerging trend in current science and technology, as they play an important role in building and driving self-powered nanodevices. To the best of our knowledge, this is the first article that introduced EVA/GO nanocomposites as a potential futuristic material for triboelectric energy harvesting application. Though graphene–polymer nanocomposites are a new attempt in TENG, Harnchana *et al.* recently reported the achievement of very high electrical output using a GO/PDMS porous-type nanocomposite.^14^ TENG is a new type of clean and sustainable energy harvesting device capable of harvesting energy from various mechanical vibrations (ocean waves, tides, raindrops and human motion).^14–17^ The working mechanism of this device is based on the contact electrification effect, where energy conversion occurs *via* surface charge transfer resulting from periodic contact between two dissimilar tribopolar materials, a triboelectric positive and a triboelectric negative material.^15–17^ Interestingly, the nanocomposite-based TENG showed great performance to the triboelectric response, with an output voltage and current 4 and 4.2 times higher, respectively, than that of the pristine EVA device. Moreover, the EVA nanocomposite TENG showed highly stable performance against severe constant cyclic load and when exposed to ambient conditions for several weeks. Finally, the novelty of this research lies in the concept of the modification of GO, whereby the large reinforcement, excellent molecular-level dispersion and high triboelectric performance of the composite can be achieved concurrently at low filler loading.

## Results and discussion

Fig. 1 illustrates the plausible chemical reactions, which occurred during the synthesis of PD–PEI-modified GO. As can be seen, dopamine can be auto-oxidized to polydopamine (PD) *via* several dehydrogenation steps, forming *o*-quinone units that can instantly cyclize to indole moieties and/or form C–C bonds. The Michael reaction takes place at the *o*-quinone moieties either at the carbonyl carbon atom or at the β-position of the inherent Michael system in an alkaline medium.^18^ This leads to the self-polymerization of dopamine and the simultaneous copolymerization with PEI. At the same time, the oxygenated groups on GO can form strong covalent and hydrogen bonds with the PD–PEI layer, making it very suitable in dispersing various solvents and polymer matrices when added.

**Fig. 2:**
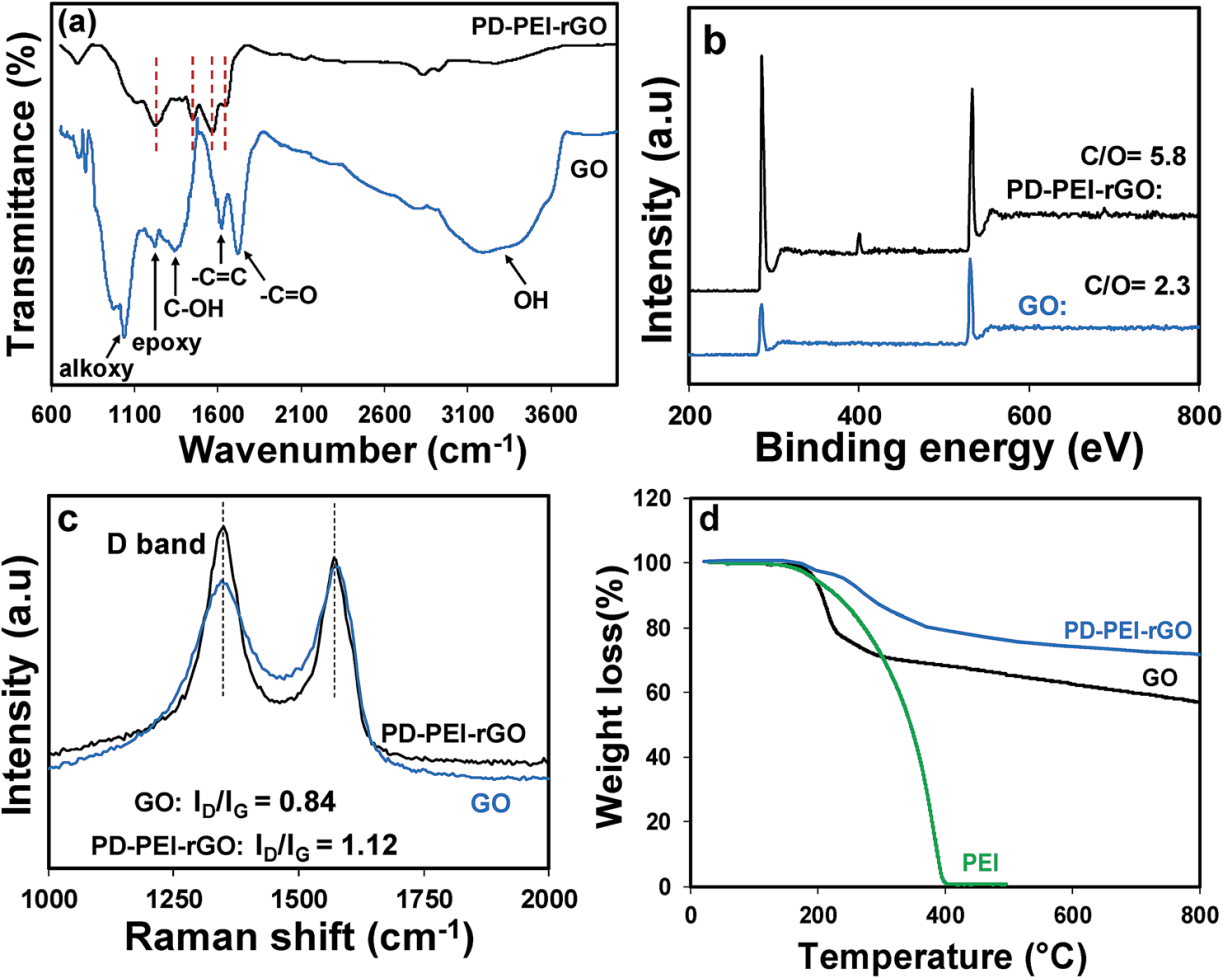
(a) FTIR, (b) XPS survey spectra and (c) Raman spectra of GO and PD–PEI–rGO; (d) TGA profiles of PEI, GO and PD–PEI–rGO.

FTIR analysis was performed first to verify the chemical changes in GO as a result of the modification process. Fig. 2a shows the FTIR spectrum of pristine GO and PD–PEI–rGO. As can be seen in the FTIR spectrum for pristine GO, owing to various oxygenated functional groups, several important characteristic peaks appeared. A broad peak for –OH stretching was observed in the range of 3200–3640 cm^−1^. Other characteristic peaks for the C=O and C=C bonds, C–OH stretching vibrations, epoxy (1224 cm^−1^) and C–O vibrations from alkoxy groups were seen at 1725, 1624, 1351, 1224 and 1039 cm^−1^, respectively.^1,10^ After modification with the mixture of PD–PEI, some noticeable spectral changes appeared in the measurement. The new absorption peaks at 2930 and 2831 cm^−1^ corresponded to the nonsymmetric and symmetric stretching vibrations of methylene, respectively, while the new peaks at 1649 and 1569 cm^−1^ represent the stretching vibrations of NHC=O, 1453 cm^−1^ for the aromatic C=C bond, and 1220 cm^−1^ for the C–N bands.^2,19^ It is important to note here that the amine groups in PEI (owing to highly branched structure) will also react with the carboxyl and epoxy groups of GO at the same time, resulting in the appearance of amide and C–N bonds in the respective spectrum. The peak at 3320 cm^−1^ can be ascribed to the N–H group of PEI. To further ascertain the attachment of PEI onto GO and to learn about the surface chemical compositions, XPS analysis was carried out. Fig. 2b shows the survey scan spectra of pristine GO and PD–PEI–rGO. As can be seen, the survey spectra of GO is composed of two peaks, C 1s (binding energy = 285 eV) and O 1s (binding energy = 531 eV), while PEI–rGO displayed an emerging new N 1s peak at a binding energy of 401 eV along with the C 1s and O 1s peaks.^20^ This result clearly endorsed the successful attachment of PDA and PEI onto the GO surface. While the pristine GO sample had C and O contents of 69.8 and 30.2%, respectively, the PEI–rGO sample showed 80.5% C, 14% O and 5.5% N contents. However, for curiosity when we checked the XPS spectra for the PD–rGO sample, only 3.7% N content was found (less than for the PD–PEI–rGO sample, results are not shown).

Next, Raman spectra were measured to understand the crystal structure, disorder and defects in the above GO specimens. As can be seen from Fig. 2c, both materials have D and G peaks at 1350 and 1581 cm^−1^, corresponding to the first-order scattering of the E_2g_ phonon of sp^2^ C atoms and structural defects induced by the surface functional groups, respectively. It is well-known that the height of the D band implies the size of the inplane sp^2^ domains.^1,3,8^ Therefore, the increase of the D peak intensity indicates the formation of more defects in sp^2^ domains in the structure. As can be seen in Fig. 2c, the *I*_D_/*I*_G_ ratio of PD–PEI–rGO is higher (1.12) than that of pristine GO (0.84), indicating that surface modification had led to an increase in the number of disorders or defects in the graphene sheets.

In order to understand the effect of modification on the thermal behavior of the GO, TGA analysis of pristine GO and PD–PEI–rGO was conducted, and the corresponding results were compared in Fig. 2d. It is apparent that pristine GO has poor thermal stability and decomposes at below 200 °C, presumably due to the loss of oxygen-containing functional groups that attach to the graphene sheets (epoxide, hydroxyl, and carboxyl groups).^1^ Following that, the decomposition, which brought about 20–22% more mass loss, was due to the thermal decomposition of the carbon skeleton. On the other hand, the modified GO showed much higher thermal stability, with only 5% weight loss at 240 °C and 28% total loss at 800 °C. The losses at above 240 °C can be attributed to the degradation of the polydopamine and PEI molecules. The TGA results also revealed that there is ∼15–16 wt% of PD–PEI in the modified graphene oxide (PD–PEI–rGO). The higher thermal stability clearly indicates that there are strong interactions between the GO and PD–PEI moieties, also endorsing the above FTIR spectrum that showed the formation of amide bonds between GO and PD–PEI due to the modification. The presence of such large amounts of PD–PEI in GO also suggests that this can be a good filler for polymer nanocomposites.

**Fig. 3:**
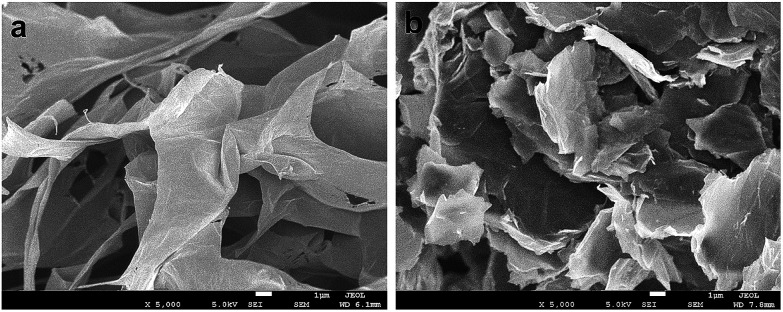
FESEM images of (a) pristine GO and (b) PD–PEI–rGO. Images were taken under 5000× magnification at 1 μm scale bar.

Fig. 3 shows typical FESEM surface morphologies of pristine GO and PD–PEI–rGO surfaces. It is apparent that before modification, the surface of the pristine GO sheets had clean and smooth surfaces and that the graphene sheets were nicely interlinked through very thin and delicate three-dimensional networks, resulting in a porous, loose sponge-like structure. However, after modification (for PD–PEI–rGO) lots of wrinkles and corrugation appeared on the surfaces. Also, some level of stacking in the graphene sheets were observed. The formation of rough and wrinkled structure clearly evidenced the covalent attachment of PD–PEI molecules onto the GO surfaces. The formation of stacking in the graphene sheets can be assumed to be due to the effects of π–π stacking interactions between the PDA–graphene sheets and interlayer hydrogen bonds between the PD–PEI layers that are covalently grafted onto the graphene surfaces.

**Fig. 4:**
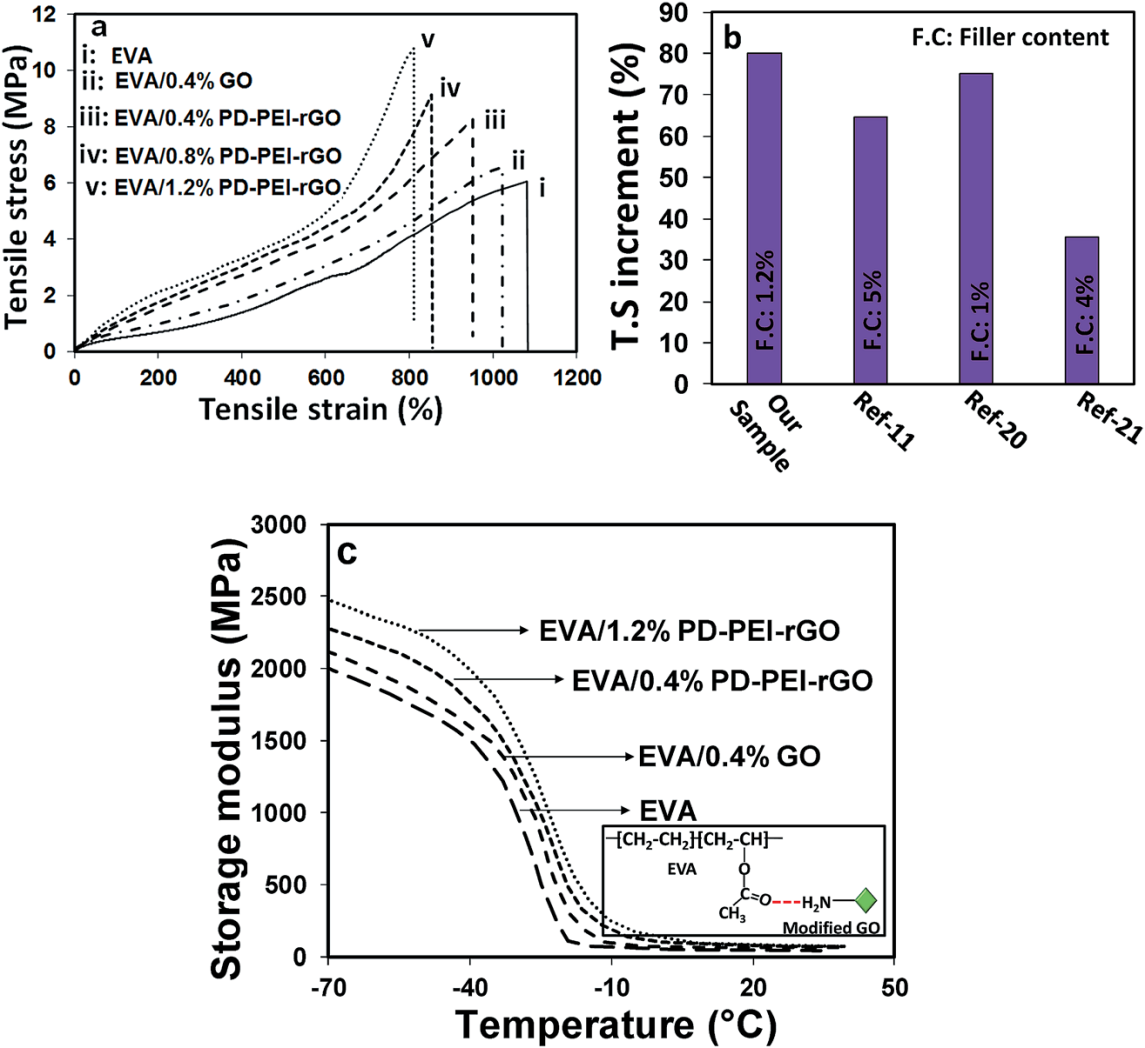
(a and b) Stress–strain curves and (c) storage modulus of various EVA nanocomposites.

To explore the potential of PD–PEI–rGO as a filler material in the area of polymer composites, they were blended with ethylene vinyl acetate copolymer (EVA) matrix to create the nanocomposites. The composites were fabricated by solution casting followed by a melt-blending process. The tensile and DMA test results of the composites will be discussed next, as they indicate the capability of the material for structural performance. Low amounts of PD–PEI–rGO (*i.e.*, 0.4, 0.8 and 1.2 wt%) were added into the EVA matrix to fabricate various nanocomposites. Fig. 4a shows the stress–strain results of various EVA nanocomposites as a function of filler content. It was observed that the addition of pristine GO into the EVA matrix (0.4 wt%) led to an increase in the tensile strength (T.S) of the composite by only 8.63% due to the inherent reinforcing ability of GO. However, dramatic enhancement in the T.S and Young's modulus (*E*) was obtained after the addition of PD–PEI–rGO into EVA, and the values increased with increasing PD–PEI–rGO content. It was interesting to see that the addition of the same amount of modified GO (0.4 wt%) led to an increase in the T.S of the nanocomposite by 4.4-fold that of the pristine GO/EVA nanocomposite.

While the T.S and *E* for pure EVA were 6.02 and 0.62 MPA after the addition of only 1.2 wt% PEI–rGO, the values increased to 10.83 and 0.93 MPa, respectively, corresponding to an increase of 80% and 50%, respectively, compared to that of the neat EVA. The above results clearly indicate that PD–PEI functionalization had effectively improved the affinity of GO for the polymer matrix. Such a large increment in the overall mechanical properties of the EVA nanocomposites can be attributed to the effects of: (i) strong interfacial adhesion between the matrix and the modified graphene nanosheets (owing to strong hydrogen bonding between the carbonyl of EVA and the amine of the PEI), (ii) effective load transfer from the polymer matrix to the graphene nanosheets, and (iii) homogeneous dispersion of the graphene nanosheets throughout the polymer matrix. The higher mechanical strength can also be explained by the greater mechanical interlocking between the EVA polymer chains and modified graphene oxide nanosheets, which possess higher surface roughness. Interestingly, these values were found to be much higher than that of many earlier reports on EVA nanocomposites (see Fig. 4b).^11,21,22^

Fig. 4c shows the storage modulus *versus* temperature curves of various nanocomposites. It can be seen that the storage modulus of neat EVA within the set temperature range (−70 to 40 °C) is considerably lower than that of the nanocomposites. After the incorporation of GO, regardless of the type (unmodified or modified), the storage modulus of the nanocomposites increased significantly. While the storage modulus of neat EVA was found to be 1981 MPa, nanocomposites containing 0.4 wt% pristine GO displayed a storage modulus value of 2160 MPa, which was ∼9% higher than that of the neat EVA. Interestingly, when the same amount of modified graphene oxide was added (*i.e.*, 0.4 wt% of PD–PEI–rGO into EVA), the storage modulus reached to up to 2320 MPa (17% higher), nearly double that of the pristine GO-containing nanocomposite. The gradual addition of PD–PEI–rGO (*i.e.*, 0.8 and 1.2 wt%) further increased the storage modulus to 2377 and 2457 MPa, which was ∼20% and 24% higher than the storage modulus of the neat EVA, respectively. Such large increments in the mechanical strengths of the EVA nanocomposites with the use of such a small amount of graphene oxide (1.2 wt%) clearly suggest the great potential of our functionalization process and that of modified GO. The tan *δ* maximum value, which corresponds to the *T*_g_ of the polymer, was also noted to increase with the addition of PD–PEI–rGO (see Table 1). The maximum increment was found to be 6.2 °C in the case of the EVA/1.2 wt% PD–PEI–rGO nanocomposite (from −30.3 °C for neat EVA to −24.1 °C). The increase in *T*_g_ for the nanocomposites clearly indicates that the free motion of EVA chains in the matrix was inhibited by the GO nanosheets due to strong filler/matrix interfacial interactions. However, this was more profound in the case of EVA/PD–PEI–rGO nanocomposites due to greater amounts of hydrogen bonding between the amine of PD–PEI–rGO and the carbonyl of EVA as a result of the homogeneous dispersion of GO nanosheets within the PVA matrix (see the inset of Fig. 4c).

**Table 1:** Tensile and dynamic mechanical results of EVA nanocomposites

Sample	Tensile strength (MPa)	Increment (%)	Young's modulus (*E*, MPa)	Increment (%)	Storage modulus (MPa)	Increment (%)	tan delta/*T*_g_ (°C)
EVA	6.02 (±1.1)	—	0.62 (±16.8)	—	1981	—	−30.3
EVA/0.4 wt% GO	6.54 (±1.3)	8.63	0.67 (±17.1)	8.0	2160	9.0	−29.7
EVA/0.4 wt% PD–PEI–rGO	8.31 (±1.4)	38.0	0.81 (±17.6)	30.6	2320	17.1	−28.4
EVA/0.8 wt% PD–PEI–rGO	9.60 (±1.3)	59.4	0.87 (±18.7)	40.3	2377	20.0	−27.3
EVA/1.2 wt% PD–PEI–rGO	10.83 (±1.4)	80	0.93 (±18.3)	50.0	2457	24.0	−24.1

**Fig. 5:**
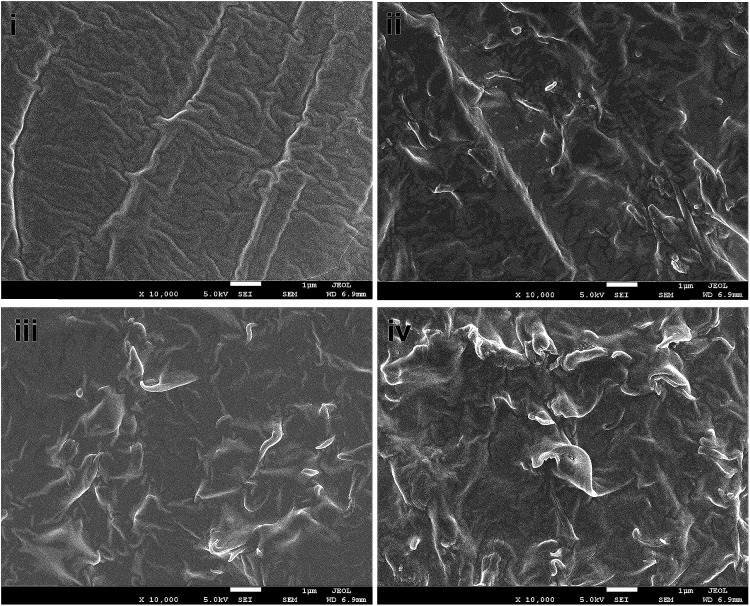
FESEM images of the tensile fractured surface of (i) raw EVA, (ii) EVA/0.4 wt% GO (iii) EVA/0.8 wt% GO and (iv) EVA/1.2 wt% GO nanocomposites.

Based on the above mechanical test data, it is necessary to examine the dispersion behavior of the PD–PEI–rGO nanosheets in the matrix; therefore, the cross-sectional surface morphology of the composites was investigated and presented in Fig. 5. The cross-sectional fracture surface of the neat EVA is shown in Fig. 5(i). It is apparent that neat EVA had a clean surface with typical fracture patterns on the surfaces. In contrast, the surfaces of the nanocomposites showed noticeable roughness. Fig. 5(ii–iv) shows the surface morphologies for EVA/0.4 wt% GO, EVA/0.8 wt% PD–PEI–rGO EVA/0.4 wt% PD–PEI–rGO and EVA/1.2 wt% PD–PEI–rGO nanocomposites, respectively. It has been observed that the surfaces of modified PD–PEI–rGO-based nanocomposites possess much higher roughness than the pristine GO/EVA nanocomposite. Moreover, the surface roughness appeared to increase with the increase in PD–PEI–rGO loadings. Compared to the pristine GO/EVA nanocomposite, the higher surface roughness for PD–PEI–rGO-based nanocomposites signifies the higher energy absorption during the tensile fracturing of the specimens due to the tighter embedment of the PD–PEI–rGO nanosheets into the EVA matrix owing to strong filler–matrix interface adhesion. Moreover, the roughness appeared all over the surfaces of the PD–PEI–rGO/EVA nanocomposites, indicating that PD–PEI–rGO were homogeneously dispersed into the matrix. Based on the above results, it can be concluded that PD–PEI–rGO is indeed a potential filler in the development of strong EVA polymer nanocomposites.

**Fig. 6:**
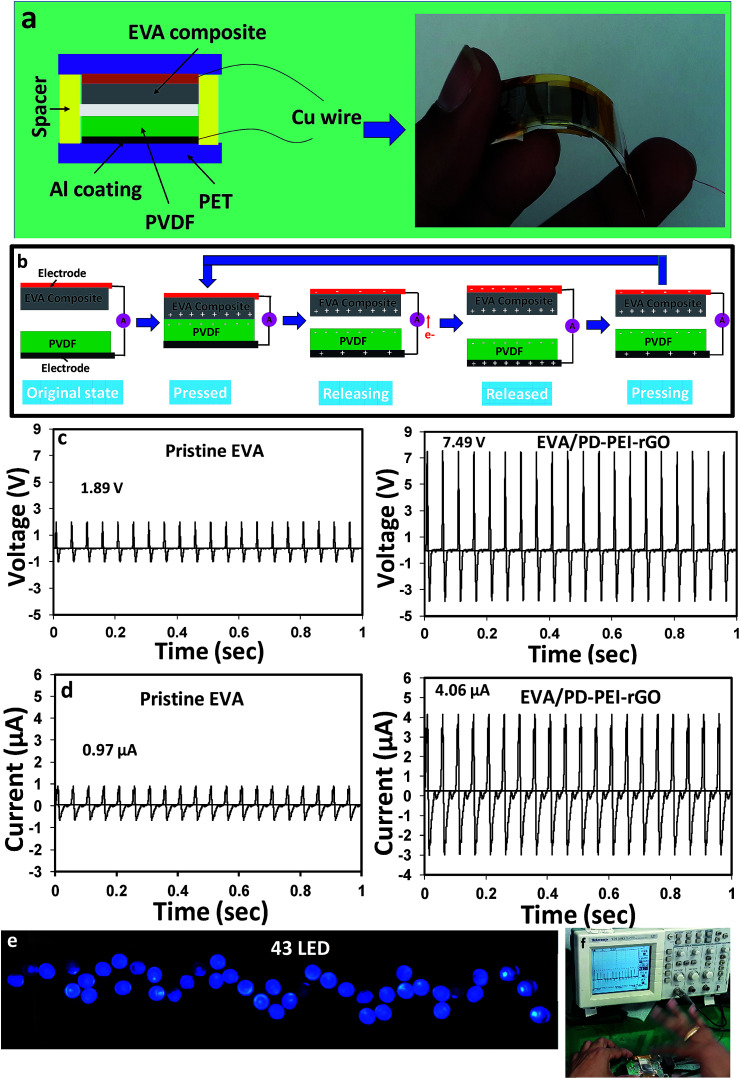
(a) Schematic diagram of the EVA nanocomposite TENG. The right-hand side image shows the real device. (b) Schematic illustration of the operating principle of the TENG device. Output (c) voltage and (d) current of said device. (e) Illumination of 43 blue LEDs. (f) The changes in output voltage *versus* time for monitoring motions by hand pressing.

To understand the multifunctional role of the EVA nanocomposite, especially for energy harvesting application, triboelectric measurements were recorded. The construction of TENG is shown in Fig. 6a. Details of the fabrication process are discussed in the experimental section. Fig. 6d illustrates the power generation mechanisms *via* TENG. Initially, there was no charge generation with no electric potential difference between the two electrodes of TENGs. When the external compressive force was applied to the device, the two insulating polymeric materials came in contact, and as a result, surface charge transfer took place at the interfaces due to the triboelectric effect. According to the triboelectric series, a PVDF film has strong negative triboelectric polarity. Due to the amine-rich surface, the EVA nanocomposite surface was believed to possess tribopositive polarity. Hence, negative and positive charges were generated on the PVDF and EVA nanocomposites surfaces, respectively. As both the materials (PVDF and EVA nanocomposite) are insulating in nature, the generated charges at each surface would, therefore, remain there (or not be quickly neutralized), leading to no electric potential difference between the two electrodes. As soon as the compression force is released, a strong potential difference between the top and bottom electrodes was created due to the electrostatic induction effect or strong dipolar difference. As a consequence, the electrons began to flow from the negative to the positive potential through the external circuit, resulting in the experimentally observed positive electric output signal (or say half of the triboelectric charge cycle) on the screen. The electron transfer occurs until an electrostatic equilibrium is established. Upon further pressing the TENG, the electrostatic equilibrium was disrupted, and the backflow of electrons took place from the bottom electrode to the top electrode until a new equilibrium was reached. This resulted in the generation of the opposite current, thereby causing reversed electrical signals to be observed. This is considered as the second half of the triboelectric charge cycle.

Fig. 6c and d compare the open-circuit voltage (*V*_oc_) and the short-circuit current (*I*_sc_) between the pristine GO/EVA nanocomposite and the PD–PEI–rGO/EVA nanocomposite-based TENGs, respectively. While the peak output voltage and current for pristine GO/EVA nanocomposites were about 1.89 V and 0.97 μA, respectively, the PD–PEI–rGO/EVA composite TENG showed electrical outputs of about 7.49 V and 4.06 μA. These values were ∼4 and ∼4.2 times higher than those of the output voltage (1.89 V) and current (0.97 μA) of pristine EVA, respectively. The higher electrical output for the PD–PEI–rGO/EVA nanocomposite TENG can be attributed to the higher surface polarity and electron-donating ability owing to the presence of the amine (from PEI) and OH groups (from DA) when rubbed with a fluoropolymer. Moreover, the embedded GO nanoplatelets in the composite can act as charge trapping sites, thereby promoting the relative dielectric property of the film, leading to an increase in the triboelectric output performance of the composite.^14^ To verify the feasibility of the above nanogenerator for practical uses, we connected 43 blue LEDs directly (in parallel) to the output of the nanogenerator without any circuit. Fig. 6e shows the digital photograph of 43 lightened blue LEDs taken after the nanogenerator was activated. The LEDs were lightened by hand pressing the TENG (see Fig. 6f) in order to see its ability for various modern portable and wireless electronics. A video of the lighting of LEDs was also presented in Video S1 (ESI†) for a better idea.

**Fig. 7:**
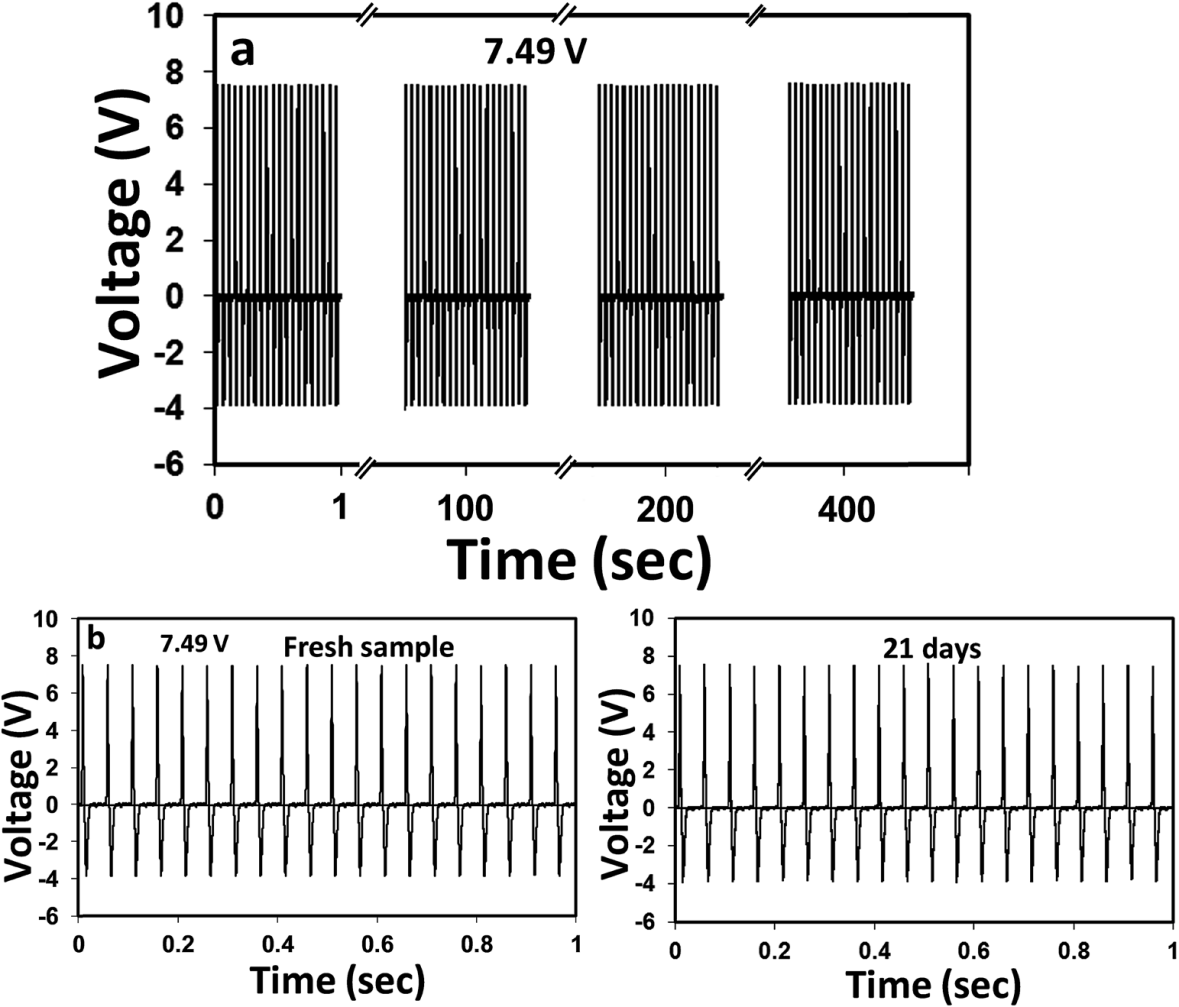
(a) Cyclic durability performance of the EVA nanocomposite TENG tested for 8000 cycles at a frequency of 20 Hz. (b) Stability test of the same device tested after immediate fabrication and 3 weeks. The device was stored under ambient conditions for 3 weeks.

Next, the cyclic performance stability and time-dependent durability of the above TENG was tested, as it determines the quality and shelf-life of the device. From Fig. 7(a), it is apparent that the device can produce absolutely stable voltage even after a continuous 8000-cyclic load. The device was also capable of reproducing the same voltage signals when tested after 21 days under similar mechanical force. These results clearly indicated that the PD–PEI–rGO/EVA nanocomposite is literally very strong and robust. Thus, no loss or deterioration in the signal was observed when subjected to severe continuous compression load. The higher Young's modulus (also indicates the rigidity) of the nanocomposite was believed to be the key factor for the above performance stability. On the basis of the above findings, it could be concluded that the EVA nanocomposite is indeed a new multifunctional nanocomposite material with great structural potential as well as able to harvest clean energy by means of the triboelectric nanogenerator. We believe that this composite can readily be used for many potential applications including street tiles, night club dance floors, floor mats and other sensing purposes where electricity can be harvested from mechanical energy, *i.e.*, dancing or jumping.

## Conclusions

We reported a novel and effective approach for GO modification to produce mechanically strong EVA nanocomposites with multifunctional capability. The surface modification was carried out by a unique mussel-inspired polydopamine-assisted Michael addition reaction. This approach showed greater promise in terms of molecular level dispersion and the interface adhesion of graphene nanosheets within the EVA matrix owing to strong hydrogen bonding interactions. As a result, the achieved polymer nanocomposites showed radical improvements in the tensile strength (80%) and storage modulus (24%) even at only 1.2 wt% of graphene content, clearly demonstrated the robustness of the functionalization process. Besides the high reinforcing capability, the addition of modified GO (PD–PEI–rGO) turned the nanocomposite into an excellent triboelectric material owing to the amine-rich surfaces. As a result, the composite triboelectric nanogenerator showed outstanding electrical responses with an output voltage of 7.49 V and current 4.06 μA, thereby lighting up the 43 blue LEDs instantaneously. Such a high output voltage also implied that our device would be very effective for large-scale industrial applications. This also suggests that the performance of the device can be amplified further by increasing the graphene content as well as the degree of amine functionalization. Considering the above fascinating achievements, it can be concluded that our functionalization strategy was indeed a versatile platform for GO modification and can hopefully attract great attention from various sectors to develop other lightweight and strong composites for wider applications.

## Experimental

### Materials

Flaky graphite powders (300 mesh) were purchased from Huadong Graphite Factory (Pingdu, China). Dopamine, Trizma base and polyethylenimine (PEI, branched) with an average molecular weight of 25 000 were purchased from Sigma Aldrich, South Korea. Ethylene vinyl acetate copolymer (EVA, Levapren 450, Lanxess) was obtained from India. Polyvinylidene fluoride (PVDF) film was purchased from Starlab Scientific Co., Ltd, Korea.

### Preparation of PEI functionalized GO (PDA–PEI–rGO)

Firstly, GO was synthesized by a modified Hummers method from graphite flakes following our earlier report.^1^ The as-synthesized GO was then thoroughly dispersed in 10 mM Tris (pH 8.5) buffer solution using a probe sonicator at 20 Hz frequency for 30 min. Next, an equal amount of dopamine (DA) and PEI (1 : 1) was added into the GO solution, and the mixture was magnetically stirred for 6 h at 50 °C. After that, the mixture was filtered by vacuum filtration and washed with large amounts of water and methanol repeatedly in order to remove any unattached DA and PEI. Then, the product was freeze-dried overnight and labelled as PD–PEI–rGO. The ‘r’ represents the partial reduction of graphene oxide.

### Preparation of PD–PEI–rGO/EVA nanocomposites

Various PDA–PEI–rGO/EVA nanocomposites were prepared by solution casting followed by the melt blending process. Briefly, PD–PEI–rGO of 100, 200 and 300 mg were dispersed separately in 25 ml of toluene by ultrasonication at room temperature for 1 h. Concurrently, EVA (25 g in 100 ml) was dissolved in toluene separately. Next, the above PD–PEI–rGO dispersions were added separately into the EVA solution and mixed thoroughly with constant stirring at 100 °C for 8 h and then cast on a glass Petri dish. After complete drying in air followed by vacuum, the above three different nanocomposite films (containing 0.4, 0.8 and 1.2% PD–PEI–rGO) were cut into small pieces and melt blended in a Haake mixture (MiniLab) at 145 °C to obtain the desired composites. Dicumyl peroxide (DCP, 1 wt%) was used as a curing agent during the blending process. The extruded strips were then made into sheets (1–1.2 mm) by compression molding in a hot press at a temperature of 150 °C with a holding pressure of ∼10 MPa for 5 min. For comparative study, a GO/EVA nanocomposite was also fabricated under identical conditions.

### Fabrication of the EVA nanocomposite-based TENG

All TENGs used in this study were vertical contact types. There were two active materials, *i.e.*, EVA nanocomposite and PVDF films, used to fabricate the device. The process of fabrication is as follows: a 1.7 cm × 1.7 cm × 1 mm piece of PD–PEI–rGO/EVA nanocomposite film was prepared. Then, the film was attached to the center of a strip of copper tape, which acted as the top electrode. The Cu tape was further attached to a PET film in order to protect the electrode from damage and as a hard supporting layer for easy handling of the devices. On the other hand, a PVDF film of the same size (but the thickness was 0.25 mm) was taken, and one side was coated with aluminum using a metal ion evaporator. This became another electrode for the device. The coated PVDF was similarly attached to a PET support. Then, the device was assembled by placing the two active surfaces face-to-face, separated by a 2 mm soft spacer and connected to the external circuit through copper wires. For comparison, a pristine EVA/PVDF TENG had also been fabricated keeping all parameters the same as earlier. It is important to note here that for the triboelectric experiment a nanocomposite of higher PD–PEI–rGO content (*i.e.*, 3 wt%) was used.

### Characterizations

The presence of surface functional groups of the pristine GO and modified GO powder were analyzed by Fourier transform infrared spectroscopy (FTIR, Bruker Optics, Billerica, USA) spectra at a scan rate of 16 with 4 cm^−1^ resolution. The surface elemental compositions of the above samples were determined by X-ray photoelectron spectroscopy (XPS) on a K-Alpha Thermo Scientific spectrometer with monochromatic Al Kα X-ray radiation. All XPS spectra were corrected according to the C 1s line at 284.8 eV. The Raman spectra of GO and PD–PEI–rGO were measured by an Alpha 300R WiTec confocal Raman microscope. The thermal stability of all specimens was tested using a TA Instruments TGA 2950 from 30 to 800 °C at a heating rate of 10 °C min^−1^ under nitrogen gas medium. The tensile tests of the EVA nanocomposites were performed at room temperature with a crosshead speed of 50 mm min^−1^. The storage modulus of the composites was measured by a dynamic mechanical analysis instrument (DMA Q800 TA) in the tension mode at a heating rate of 5 °C min^−1^. The surface morphology of the tensile-fractured samples was observed by field emission scanning electron microscopy (FESEM, JEOL 6400F) after coating with platinum. Triboelectric performance of the TENGs was measured as follows: the device was fixed onto a metal zig, and a 20 Hz square wave input was applied to the device from a shaker (the Modal Shop K2004E01), which was controlled by a function generator (Keysight 33220A). The bottom electrode was pressed up by a shaker with 14 N force, while the top electrode was fixed onto a lab bench. The input force was measured by a force transducer (Brüel & Kjær 8230-001). The output voltage and current data were collected using a pulse analyzer (Brüel & Kjær 3560-B-030) and PC.

## Conflicts of interest

We have no conflict of interest.

## Supplementary Material

NA-001-C9NA00142E-s001
